# Odor Detection in *Manduca sexta* Is Optimized when Odor Stimuli Are Pulsed at a Frequency Matching the Wing Beat during Flight

**DOI:** 10.1371/journal.pone.0081863

**Published:** 2013-11-21

**Authors:** Kevin C. Daly, Faizan Kalwar, Mandy Hatfield, Erich Staudacher, Samual P. Bradley

**Affiliations:** Department of Biology, West Virginia University, Morgantown, West Virginia, United States of America; Barnard College, Columbia University, United States of America

## Abstract

Sensory systems sample the external world actively, within the context of self-motion induced disturbances. Mammals sample olfactory cues within the context of respiratory cycles and have adapted to process olfactory information within the time frame of a single sniff cycle. In plume tracking insects, it remains unknown whether olfactory processing is adapted to wing beating, which causes similar physical effects as sniffing. To explore this we first characterized the physical properties of our odor delivery system using hotwire anemometry and photo ionization detection, which confirmed that odor stimuli were temporally structured. Electroantennograms confirmed that pulse trains were tracked physiologically. Next, we quantified odor detection in moths in a series of psychophysical experiments to determine whether pulsing odor affected acuity. Moths were first conditioned to respond to a target odorant using Pavlovian olfactory conditioning. At 24 and 48 h after conditioning, moths were tested with a dilution series of the conditioned odor. On separate days odor was presented either continuously or as 20 Hz pulse trains to simulate wing beating effects. We varied pulse train duty cycle, olfactometer outflow velocity, pulsing method, and odor. Results of these studies, established that detection was enhanced when odors were pulsed. Higher velocity and briefer pulses also enhanced detection. Post hoc analysis indicated enhanced detection was the result of a significantly lower behavioral response to blank stimuli when presented as pulse trains. Since blank responses are a measure of false positive responses, this suggests that the olfactory system makes fewer errors (i.e. is more reliable) when odors are experienced as pulse trains. We therefore postulate that the olfactory system of *Manduca sexta* may have evolved mechanisms to enhance odor detection during flight, where the effects of wing beating represent the norm. This system may even exploit temporal structure in a manner similar to sniffing.

## Introduction

Olfaction, like other sensory modalities, must detect and represent sensory cues within the context of their transient and temporally structured nature. The temporal structure of odor plumes (or trails) as experienced by an animal arises from three sources: the discontinuous and dynamic nature of the odor plume, odor-guided locomotory behaviors, such as casting and zigzagging, and active odor sampling behaviors, including sniffing and antennal flicking (e.g. 1–3). Wing beating is another behavioral mechanism that imposes temporal structure on olfactory stimuli and could impact olfactory perception. Like sniffing, details of wing beating such as wing beat frequency, orientation and the trajectory of the wing as it passes by the antennae, are all dependent on the behavioral context [4]. For example, low speed hovering flight such as when a moth looses track of the plume, or as it feeds on the wing, brings the path of the wing in closer proximity with the antennae thereby increasing its effect. Furthermore, biomechanical and modeling studies of insect wing beating in plume tracking moths demonstrate that this behavior causes oscillations in the airflow around the antennae of both the non-flying silkworm moth [5] and the sphinx moth, Manduca sexta [6,7], which tracks plumes on the wing. In the case of silkworm moths, results from a dynamically scaled model suggested that wing-beating increases airflow leakage between the sensillum by as much as 500% [4]. Empirical studies confirming this however, are still lacking. 

These findings raise the question of whether or not the temporal structure induced on odor plumes by the beating wings could impact behavioral measures of odor detection. Recently, we found that odor presented in pulse trains at frequencies replicating a beating wing readily results in a pulse tracking response [8]. This was observed in electroantennogram (EAG) and antennal lobe (AL) local field potentials (LFP) recordings as frequency-matched oscillations, and from multiunit spiking responses, in which cells recorded from within the AL produced discrete bursts in response to each pulse of the pulse train. Both LFP and unitary spiking measures tracked pulses beyond the maximum wing beat frequency, suggesting that *Manduca* has evolved to track temporally complex stimuli with extraordinary resolution. Furthermore, power spectral density analysis revealed very narrow band power (+/- 2 Hz) at the pulsing frequency indicating that the AL tracks the temporal structure with far greater fidelity than had been previously described [9,10]. We also found that bath application of the GABA_A_ receptor antagonist bicuculline resulted in a complete loss of pulse tracking supporting previous findings that local inhibitory network processes mediate tracking [9]. Finally, preliminary psychophysical results suggested that moths were better able to detect a target odor when pulsed as opposed to presented continuously. 

However, in order to normalize the total amount of odor delivered in both the pulsed and continuous stimuli, pulse trains were presented for a relatively longer duration. Thus the interpretation of these prior results rests on assumptions about how stimulus duration and sensory integration time affect sensory perception. For example, does total stimulus time, independent of temporal structure (i.e. whether pulsed or continuous), affect the likelihood of eliciting a conditioned response? In addition, does pulse train duty cycle (i.e. the ratio of odor ‘on’ to ‘off’ per pulse cycle) affect detection measures? Indeed, longer pulses have been associated with poorer pulse tracking performance in EAG [11] and antennal lobe [10] recordings. Finally, there are other pulse train parameters, such as how the odor is pulsed, that remain unexplored. Prior research for example presented odor pulses that were interleaved with pulses of clean air in order to maintain a constant flow from the odor delivery system. However, the wing beat causes an oscillation in air flow velocity [6] similar to mammalian sniffing but it remains unclear if oscillating velocity maters. To address these issues we performed several experiments, which systematically varied different pulse train parameters while maintaining all pulse trains at 20Hz. Our results establish that the velocity of the stimulus and its duty cycle affect measures of detection in a manner consistent with the flux detector hypothesis [12]. Puffing odors, that is presenting individual pulses of odor not interleaved with clean air pulses, did not enhance sensitivity indicating that the overall velocity at which odors pass the antennae is important but not the oscillating velocity of the flow per se. However, the most striking finding is that in the absence of odor, “blank” pulse trains generally produce significantly fewer responses than a continuous blank. This significant “false positive response” to continuous blanks may be attributable to non-olfactory cues such as mechanosensory stimuli. 

## Methods

### Conditioning and testing apparatus

The odor delivery stage consisted of a custom exhaust and a stimulus control system. A 13x13 cm exhaust port was positioned at the back of the stage. During conditioning and testing, each prepared moth (see below) was placed such that its head was in the center of this port, directly in front of the opening. After each stimulus passed over the moth, the exhaust captured and removed effluent from the odor cartridges. Exhaust flow was calibrated using a hotwire anemometer (Traceable Hot Wire Anemometer; Fisher Scientific) to produce an ambient airflow of ~30 cm/s where the moths head was positioned. 

**Figure 1:**
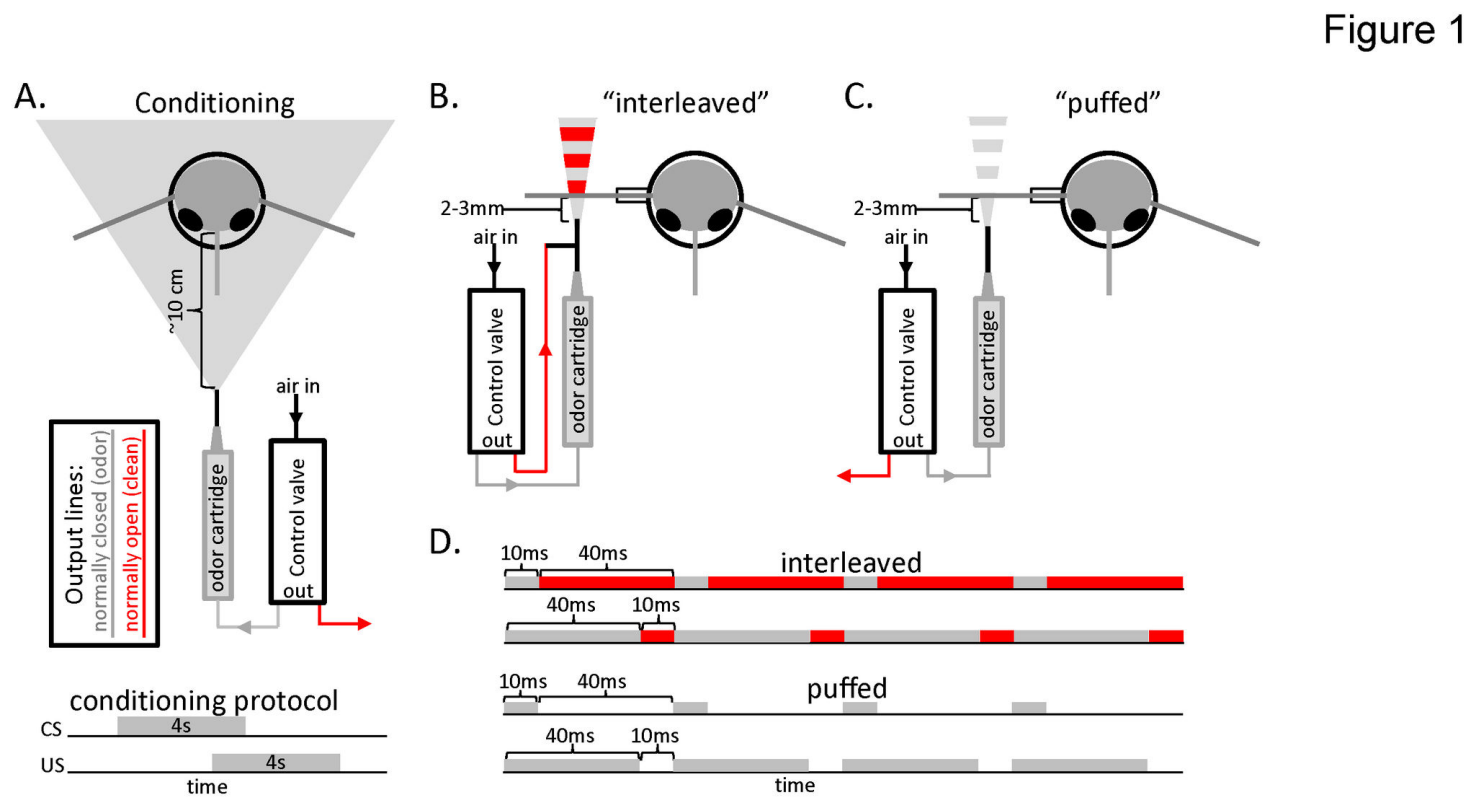
Schematic of the olfactometer configuration and stimulation protocols for conditioning (A) and testing (B-D). **A**. For Pavlovian conditioning of all moths, the odor cartridge was connected to the normally closed output line (in gray) and the normally open output line (in red) was not used. The nozzle was placed 10 cm from the moth to provide enough distance to create an odor dispersion field (inset gray triangle) wide enough to cover most of the antennae. During each conditioning trial, odor (conditioning stimulus) was presented for 4 s; 3 s into odor presentation, the moth was presented with 0.75 m sucrose solution (unconditioned stimulus). **B**. In experiments 1-4 and 7 conditioned moths were tested with pulsed odor that was interleaved with clean air. This was achieved by connecting the normally open output line (red) with to the output of the normally closed line (gray) after the odor cartridge. In this configuration air is constantly flowing out the nozzle (inset gray and red triangle), independent of odor. **C**. In order to create oscillations in air flow velocity the normally open output line was not used (as in A). In both B and C the odor delivery nozzle was positioned approximately 2-3 mm in front of a single antenna, which was held in position at its base. This insured that the temporal structure of the pulse train was preserved and that further dilution of the odor with the surrounding air was minimized. **D**. Stimulation protocols used during testing. Shown are the first 200 ms (4 pulses) of the 4 s 20 Hz pulse trains for both duty cycles and stimulation protocols (interleaved and puffed).

The olfactometer was supplied via a central air line. Air was dehydrated with a 500 cc Drierite cartridge (Indicating Drierite, mesh 8; Drierite: 23025) then filtered through a 500 cc charcoal filter made from a Drierite cartridge and using 20–60 mesh activated charcoal (Sigma-Aldrich: C3014) to remove organic compounds. Air flow through the olfactometer was controlled via a flow meter (Cole-Parmer: 1-010293). Cleaned air then passed into a three-way valve (Lee Co.; LFAA1200118H) which was controlled by a programmable logic chip (Direct logic; PLC5). As shown in Figure 1 we used three olfactometer configurations to control the flow of odor from the olfactometer nozzle to the moth antennae. For conditioning to the target odor, we presented odor as single 4 s continuous pulses from the odor nozzle from a distance of 10 cm. The output of the olfactometer was set to an outflow velocity of ~375 cm/s (Figure 1A). This distance and velocity is our standard configuration, which allows the undiluted odor to form a plume of sufficient dispersion to cover most of the antennae and thereby insures that the moth receive and hence learn the odor-food relationship [13]. In this configuration, air flowed into the inlet port of the stimulus control valve then immediately out a “normally open” outlet port and away from the stage. In order to provide odor stimulation, the valve was activated resulting in air being shunted from the normally open to the “normally closed” outlet port. The normally closed port was connected to an odor cartridge via 1.58-mm ID Tygon tubing. On the other end of the cartridge was a 1.5 mm ID nylon nozzle from which odor exited the system. 

During the testing phase of most experiments, odor laden and clean air were “interleaved” to create pulse trains where the velocity of the flow was held approximately constant during stimulation. As shown in Figure 1B, this was achieved by simply connecting the outputs of both the normally open and normally closed output ports to two arms of a 1.5 mm ID nylon T-fitting. In this case the third arm of the T served as the output nozzle. Finally, in a subset of experiments odor was “puffed” on the antennae in a manner that directly caused an oscillation in airflow velocity to approximate the changing velocity that occurs with wing beating [6]. As shown in Figure 1C the normally closed output port connected directly to the odor cartridge with a 1.5 mm ID nylon nozzle for odor delivery and the normally open port was not used. 

In addition to puffing versus interleaving odor and clean air, we also varied pulse duty cycle. That is, we varied the ratio of time during each 50 ms pulse cycle that odor was being delivered. Two duty cycles were used; 10:40 ms and a 40:10 ms. Thus, in all cases odor was pulsed at a frequency of 20 Hz (i.e. ever 50 ms). A schematic of output from the olfactometer nozzle as a function of interleaving for the two duty cycles is provided in Figure 1D as well as a comparison of the difference between interleaving and puffing.

Odor cartridges were made from stock borosilicate glass tubing with barbed nylon fittings. Each cartridge had a volume of 1.7 ml and odorant was placed into the cartridge on a piece of #1 Whatman filter paper. Three different odors were used in this experiment: racemic linalool (LOL; Sigma, 97% pure), 2-hexanone (HEX; Sigma, 98% pure), or 1-hexanol (HXL; Sigma, 97% pure). Moths were always conditioned using 3 µl of undiluted odor. Subsequent testing with the conditioned odor was with one or all concentrations from a 4 log step dilution series (0.001-10 µg in 2 µl light mineral oil). During all testing, the olfactometer nozzle tip was placed by micromanipulator ~2-3 mm from the leading edge of the right antenna and approximately in the center of the length of the antennal flagellum (Figures 1B, C). This distance minimized further dilution of the odor and breakdown of the temporal structure of the pulse train [14]. For testing, dilutions were made approximately 30 min prior to testing and each cartridge was used only once to insure consistent delivery of specific concentrations [14].

### Validating stimulus dynamics and confirming sensory input

The basis of our olfactometer is the Lee Co. 3-way valve, which is commonly used in olfactory research (e.g. 15–18). However, there has been relatively little effort to characterize odor delivery with these valves. Furthermore, the different stimulation configurations were designed to produce different flow effects but the actual shape of these stimuli is unknown. Therefore, we first characterized the time varying structure of the pulse train stimuli produced by the olfactometer. Specifically, we used anemometry, photoionization detection (PID), and electroantennograms (EAG; respectively) to: 1) quantify the non-olfactory, flow and mechanical effects from olfactometer valve actuation; 2) establish that when odor was pulsed at 20 Hz, odor concentration oscillated at that frequency; and 3) confirm that the moths antenna could track odor pulsed at these frequencies. For all of these measures, we placed the respective measurement sensor in the same position on the odor delivery stage, directly in front of the exhaust port; this was the same position as the moth’s antenna in the behavioral tests (~2-3 mm downwind of the olfactometer nozzle). These studies were performed separately from each other and from the behavioral experiments as measurement accuracy is impacted by the presence of the moth’s antennae and/or other measurement probes. We replicated both duty cycles and olfactometer flow rates used in the behavioral tests. Finally all three measures were analyzed using power spectral density (PSD) analysis to determine frequency content from 1-150 Hz; this was performed using NeuroExplorer software.

Anemometry measures were made with a MiniCTA anemometer (model 54T30; Dantec Dynamics, Denmark) using their miniature wire probe (model 55P16). This sensor can measure the small flows our odor delivery system produces and can accurately measure fluctuations in flow velocity as low as 5.0 cm/s. Positioning the sensor in the ~30.0 cm/s background flow of the exhaust port, resolves issues of sensor reliability due to convection effects at low flow rates. Anemometer signals were digitized at 30 kHz using a Digidata 1440A A/D converter and Axioscope data acquisition software (Molecular Devices, Sunnyvale, California).

**Figure 2:**
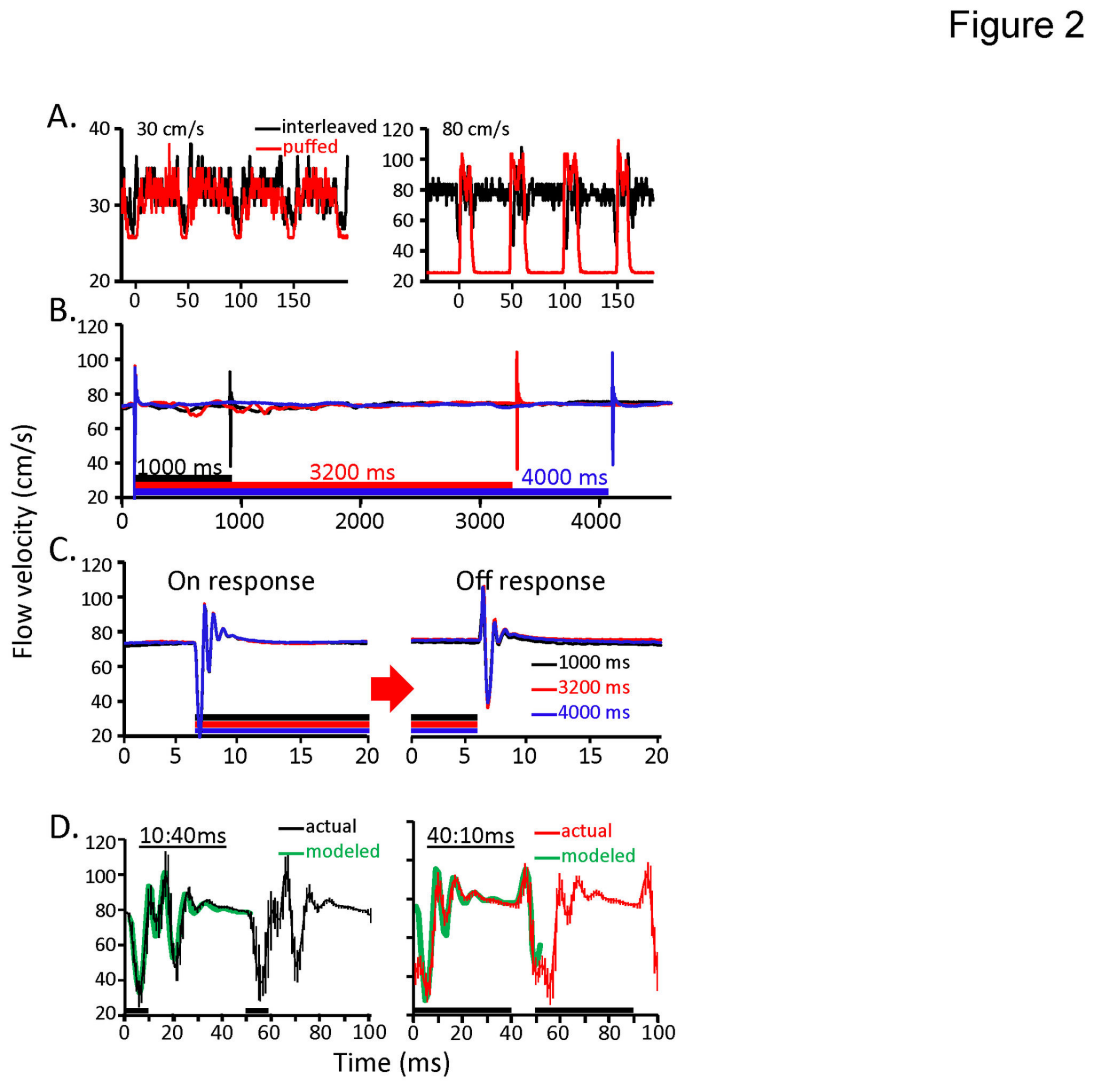
Hotwire anemometry establishes that odor valve actuation causes stereotypic flow artifacts. **A**. Raw hotwire anemometry traces in response to four consecutive pulses of odor using both the interleaving (black traces) or puffing (red traces) and an olfactometer flow of 30 cm/s (left panel) or 80 cm/s (right panel). Left and right panel also display 40:10 ms and 10:40 ms duty cycles respectively. Note that in all cases the cycle rate is on a 50 ms time scale (i.e. a pulsing frequency of 20 Hz). **B**. Three continuous stimuli of differing durations: 800 ms (black), 3200 ms (red), and 4000 ms (blue). Traces shown are averaged across 3 repeats each to eliminate noise associated with measurement error of the Mini CTA probe (see **A** for raw traces). Inset colored bars indicate stimulus duration for corresponding colored traces. **C**. Close up and aligned view of on and off response artifacts shown in B. D. Mean anemometry response over two pulse cycles (pulse durations are inset black bars along x-axis). Results based on averaging 40 consecutive 100 ms samples from a 4 s 20 Hz pulse train. Inset green trace represents the predicted integration of the on and off artifacts shown in B. Error bars are ±1 SD. Note that both duty cycles produce a unique anemometry artifact profile. Nevertheless, these profiles are accurately described as a simple linear summation of the on and off artifacts shown in **B**.

First, in Figure 2A we show raw anemometry measures from odors that were either puffed or interleaved with clean air. As examples the 40:10 ms duty cycle is shown for the 30 cm/s nozzle flow (left panel) and the 10:40 ms duty cycle is shown for the 80 cm/s (right panel). Note that in both cases the flow for the puffed stimulus oscillates at the 20Hz pulse rate, but in a duty cycle specific manner. The flow of the interleaved stimulus however maintains a mean flow that is approximately equal to the velocity of each puff particularly for the 80 cm/s flow where the ambient flow of the exhaust (~30 cm/s) is well below the flow of the olfactometer. This indicates that the odor is hitting the antennae in both configurations at approximately the same velocity. However, whereas puffing quickly drops back to the background airflow velocity of ~30 cm/s the interleaving does not. Interleaving does however produce a flow artifact that is particularly noticeable at 80 cm/s. To characterize this artifact we presented 3 different duration continuous stimuli to determine whether this artifact is caused by the valve opening or closing, or both. Figure 2B displays anemometer traces from the three different durations of continuous stimuli used in the behavioral studies (see Table 1). All stimuli are aligned by stimulus onset and are an averaged measure of three repeats to eliminate noise from measurement error (as seen in Figure 2A). This panel shows that as the valve actuates (to either the opened or closed state) there is a flow artifact, resulting in brief and rapid increases and decreases in flow velocity. Closer inspection of the time aligned on and off actuation effects (Figure 2C) indicates that the opening and closing of the valve produces distinct ‘ringing’ artifacts in flow velocity that last for a total of ~3 ms. The effect of opening the valve resulted in an initial drop in flow lasting for ~1 ms followed by an amplitude modulating oscillation around the mean flow rate. The effect of closing the valve results in an initial 1 ms duration increase in flow rate, again followed by an amplitude modulating oscillation in flow. 

**Table 1:** List of experimental groups and parameters that were varied in each.

Experimental group		Odor (CS)	Duty Cycle (ON:OFF ms)	Continuous Duration	Test Velocity	Pulse Method	N
Exp1		HEX/HXL/LOL	10:40	800 ms	80cm/s	interleaved	180 (60/odor)
Exp2		HEX/HXL/LOL	40:10	3200 ms	80cm/s	interleaved	180 (60/odor)
Exp3		HEX	10:40	4000 ms	30cm/s	interleaved	60
*Exp4		HEX	10:40	4000 ms	30cm/s	interleaved	60
Exp5		HEX	10:40	800 ms	30cm/s	puffed	60
Exp6		HEX	10:40	800 ms	80cm/s	puffed	60
**Exp7		HEX	10:40	800 ms	80cm/s	interleaved	150(30/con.)

All pulsed stimuli were at 20 Hz.

* Exp4, the continuous stimuli were 1/5^th^ concentration.

** Exp7, moths were only tested with a single concentration (con.) for both stimulation protocols.

**Figure 3:**
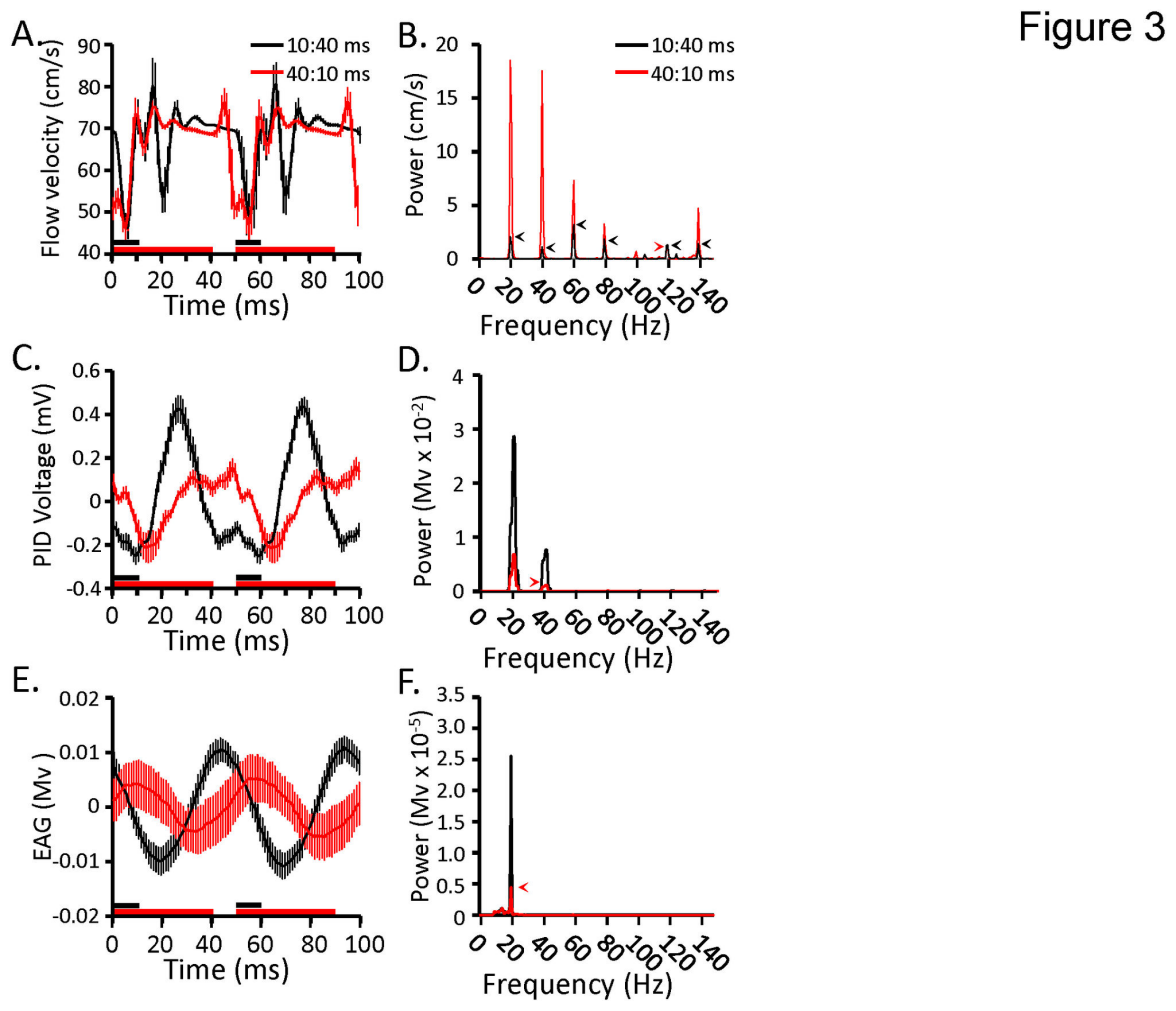
Three measures of olfactometer output demonstrate that the 20 Hz odor pulse trains are preserved through primary olfactory input but the valve artifacts are not. **A**,**C**,**E**. Mean response over two pulse cycles based on averaging 40 consecutive 100 ms samples from a 4 s 20 Hz pulse train for the two duty cycles: 10:40 ms (black), and 40:10 ms (red). Results from hotwire anemometry (same as in Figure 2D; **A.**); photo ionization detection (C.); and electroantennogram (E.). Inset horizontal lines indicate pulse duration and are color coded to correspond to individual traces. Error bars are ±1 SD. **B,D,F**. Corresponding power spectral density (PSD) analyses showing the amount of oscillatory power as a function of frequency for the hotwire anemometry (**B**); photo ionization detector (**D**); and electroantennogram (**F**). Individual traces are color coded (as above) to correspond to the two duty cycles. In cases where the peak in the power spectra for a given duty cycle was occluded we highlight it with like colored arrowheads. Note that there are several spectral peaks in the anemometry results that are greatly reduced or absent in the PID and EAG results.

In order to determine the relative consistency of the pulse response when presenting odors as pulse trains, we segmented 4 s pulse trains into forty 100 ms windows (two 50 ms pulse cycles in duration), and averaged the response for every two pulse cycles. As shown in Figure 2D, both duty cycles produced a unique flow velocity profile. Since the on and off valve artifacts are collectively ~7 ms in duration, it was likely that these artifacts integrate (i.e. interfere) differently for the 10:40 ms and 40:10 ms duty cycles. We therefore modeled the interaction of the on and off artifacts for both duty cycles as a linear interference of the two overlapping effects, using the raw traces shown in Figure 2C; these results are inlayed in each panel for the first pulse (inset green trace). This close match of the model to the first pulse supports the conclusion that each duty cycle produces a unique flow velocity profile that simply represents the degree of overlap of the on and off artifacts. Finally, in order to compare anemometry with both the PID and EAG, the results displayed in Figure 2D are presented as a single panel (Figure 3A). In addition, to compare frequency content of the pulse trains, the power spectral density (PSD) for both duty cycles is presented in Figure 3B. The PSD results indicate that there are narrow band spikes in power at 20 Hz (the pulsing frequency) and several additional frequencies. 

While anemometry quantifies the flow characteristics of the olfactometer, it does not provide information about the temporal structure of the odor stimulus. In order to measure the relative odor concentration over time, a fast response photo ionization detector (PID; 200A-miniPID; Aurora Scientific) was used with the standard 10.6 eV lamp. With a frequency response of 330 Hz, the miniPID can track time varying concentration caused by interleaving clean and odor laden air. The sensor tip was placed perpendicular to the olfactometer nozzle. The PID vacuum pump was set to low and the signal was internally amplified by 1x. The signal was then passed to a Neuralynx AC amplifier, amplified 500x and digitized at 30 kHz (Cheetah 32; Neuralynx). Only 2-hexanone was used for this validation, which has an ionization potential of 9.44 eV.

As shown in Figure 3C, the two duty cycles produced clear evidence of an oscillating voltage from the PID, which translates to an oscillation in odor concentration, though there are differences between them. In addition, there are smaller fluctuations in the voltage measures. By overlaying and precisely aligning these smaller fluctuations to the anemometer artifacts, we observed a close correspondence between the two measures with only a 5 ms linear phase lag from when the anemometer artifacts occurred to when they emerged in the PID measures (not shown). Unlike the anemometry results, the PSD analysis of the PID data indicates very little frequency content outside 20 Hz, but a small amount of 40 Hz content remains (Figure 3D.) 

Finally, EAG recordings were made by first excising a single antenna from male and female moths. A straight edge razor was used to produce clean cross sectional cuts ~2 mm from both the base and distal tip of the flagellar segment. The prepared antenna was affixed to an EAG probe with conductive gel (Spectra 360; Parker Laboratories). The EAG signals were passed through an IDAC-2 DC amplifier (Syntech, NL) at unity gain and passed to a Neuralynx AC amplifier and amplified 500x and digitized at 30 kHz. This intermediary “passing” of the EAG signal was performed because the A/D sampling rate of the Syntech system (100 Hz) was too slow to accurately capture the temporal details of the 20 Hz oscillations produced by pulsing. 

Figure 3E represents the mean pulse response for the EAG traces. In this case, both duty cycles produced an oscillating signal that approximated a sine wave. Consistent with this observation, PSD analysis identified power in a single 20 Hz band (Figure 3F) indicating that only the 20 Hz frequency content was present in the EAG trace. However, we note that the lack of temporal structure beyond 20 Hz in the EAG more likely reflects the resolution of the EAG measure. Overall, these results indicate that the olfactometer does provide discrete pulses at 20 Hz that the antennal sensory cells are able to track. Furthermore, valve actuation causes artifacts, which could provide moths with an additional non-olfactory sensory cue. It is also worth noting that the two duty cycles produced relatively different amplitude signals in the PID measures, which correspond to differences in amplitude observed in the EAGs. This difference in amplitude did not appear to be the results of cartridge depletion but rather the relatively smaller amplitude signals for the 40:10 ms duty cycles is attributable to less time for the odor to clear the system between pulses.

### Subjects

*Manduca sexta* were reared in-house using standard diet [19] and rearing methods (eg. 8). At pupal stage 17, pupae were sorted by sex, and then placed individually into brown paper bags. Bags were placed in an incubator (Percival I-66LLVL; Aimes, Iowa) on a 16:8 reverse light:dark cycle. Temperature was maintained at 25°C and relative humidity at 75%. Pupae were checked daily at the beginning of the dark cycle to identify and date those individuals that had eclosed. Eclosed adult moths were held in the chamber for 5-7 days post-eclosion to ensure both complete olfactory system development and feeding motivation. Roughly equal numbers of males and females were used in all experimental groups.

### Preparation for behavioral experiments

Moths were prepared using our standard protocols [13]. Briefly, individual moths were inserted into an aluminum tube (1.27 cm ID, 4 cm long) and restrained with pipe cleaner and tape leaving only the head exposed while restricting the moth’s movement. The tube was then attached onto an aluminum base. The proboscis was extended and threaded through a 4 cm length of 1.58 mm ID tubing (Tygon; Cole–Parmer, Vernon Hills, IL) such that the distal tip (~1.5-3 cm) was exposed and unrestrained. The Tygon tubing was then attached to the aluminum tube with soft dental wax. A Teflon coated silver electrode (125 µm diameter) was placed in the right side of the head capsule between the sagittal mid-line and the right compound eye, bringing it into physical contact with the cybarial pump muscle. The cybarial pump muscle is the largest feeding muscle in the head capsule and its activation is used as our primary indicator of feeding activity. A reference electrode, made of the same silver wire, was inserted into the contralateral eye. Electrodes were connected to wire leads attached to the aluminum tubing, which were plugged into wiring on the base. This finished preparation could then be connected to an amplifier (DAM 50; WPI Inc.). Output from the amplifier was fed into an oscilloscope and a loudspeaker, which were used by the trained observer to score behavior.

### Pavlovian Conditioning

Pavlovian conditioning was used to establish a conditioned behavioral response (CR) to target odorants. This conditioned behavior could then be used as a psychophysical assay of odor detection as a function of pulsing. Here, moths were presented with six forward-paired conditioning trials with one of the three odorants (see Fig. 1A). For each conditioning trial the odorant (conditioned stimulus; CS) was presented to the antennae for four seconds. Three seconds into the odor stimulation the unconditioned stimulus (US), a ~5 µl droplet of 0.75 M sucrose solution, was presented to the exposed tip of the partially unfurled proboscis; US exposure lasted 4 sec. Thus, there was a 1 s CS-US overlap. Each moth was trained and tested with a single odorant (see Fig. 1A for a schematic depiction). During conditioning, a response was recorded if the moth exhibited increased activity of the feeding muscle as observed on the oscilloscope and/or loudspeaker, and/or if the moth extended its proboscis. These responses had to occur in the first 3 s of the CS presentation (prior to US presentation) in order to be identified as a CR. Responses were scored as 0 for nonresponsive and 1 for a response; this method of data collection correlates very highly with physiological measures of change in feeding muscle activity associated with learning [13]. Finally, within experiments, multiple observers collected data to minimize experimenter bias.

### Behavioral tests of conditioned response

To evaluate the effect of pulsing odor on odor detection relative to continuous stimulation, all moths in all experimental conditions were tested with both pulsed and continuous stimuli. Moths were tested at both 24 and 48 hours after conditioning. Within a test day, each moth received either pulsed or continuous stimuli but never both. Half of all moths received pulsed stimuli on day one and continuous stimuli on day two, the second half received the reverse; this was randomized to control for any sequence and days effects. Moths were tested with the CS odorant only. By contrast to the conditioning phase, during testing, moths were given 7 seconds to respond, but scored in the same way. During testing, whenever the CS was presented at the 5 different concentrations, they were always presented sequentially from low to high to minimize non-associative effects such as sensory adaptation. In one experiment, moths were tested with the blank and then a single concentration to assess these potential non-associative effects. Prior to presentation of any odor, a blank (no odor) was presented to establish a baseline false positive response rate to non-olfactory components of the stimulus. The blank was presented as either a pulse train or a continuous stimulus and was matched to the corresponding stimulation protocol used for odor stimuli on that day. For all experiments requiring multiple test stimuli within a day, a 6 min inter stimulus interval was used to further minimize any non-associative effects.

To precisely control stimulus delivery during testing, the CS was presented to a single antenna. This antenna was extended and held in position by slipping it through a small wire coil spring (5 mm long; 3 mm ID) that attached to the side of the aluminum tube (see Figure 1). This held the antenna perpendicular to the moth’s body. This antennal preparation was set up at least 30 min prior to testing, where it remained until the end of the second day of testing. 

### Experimental design for behavioral studies

Our previous psychophysical studies of pulsed odor stimulation suggest that the olfactory system of *Manduca* is more sensitive to odor when pulsed at frequencies consistent with the wing beat, which is in the range of ~18-28 Hz [8]. The goal of the current study was four-fold. The first goal was to establish whether features of the pulse train, specifically the on:off duty cycle, the olfactometer outflow velocity, and differences in pulsing method, affect measures of odor detection. Second was to test the generality of prior results across different odors. The third goal was to determine if the differences in the total stimulus duration, between the stimuli in our experiments, could account for increased sensitivity to pulsed odor. Finally, we wanted to determine if non-associative effects from repeated test stimulations across the dilution series, such as sensitization, adaptation and/or extinction, affected odor detection measures. To achieve these goals we designed seven different experiments (Table 1) where moths were first conditioned to a target odor then subsequently tested with the CS as both 20 Hz pulsed stimuli and continuous stimuli. The design of the experiments was such that data could be compared across experimental groups in different combinations to test specific hypotheses.

First, to determine which features of the pulse train affected measures of detection, experimental groups 1 and 2 were conditioned and tested with one of three different odors as the CS: LOL, HEX, HXL. Each odor was used in separate sub-groups of moths. Pulsed odor was presented as a 4 sec pulse train with either a 10:40 ms (Table 1, Exp1) or a 40:10 ms (Table 1, Exp2) on:off duty cycle. This allowed us to simultaneously assess the effects of duty cycle on conditioned response (CR) measures across different odors. The continuous stimulus for each duty cycle was matched to the total integrated ‘on-time’ of the pulse train (800 ms and 3200 ms for the 10:40 ms and 40:10 ms duty cycles respectively). 

Experimental groups 1 and 2 provide the same integrated odor-on time for both stimulus protocols, presenting the moths with the same approximate amount of odor. However, the total duration of the pulse train was considerably longer than the continuous stimuli (i.e. 4000 ms pulse train vs. 800 ms and 3200 ms continuous stimuli for experiments 1 and 2 respectively). Hence spreading the odor stimulation over a longer period of time could result in a lower concentration at the level of integrated sensory input. To determine if the differences seen between the pulsed and continuous stimuli were related to the differences in their total durations, we presented both stimuli for the same total time (4000 ms) in two separate experimental groups. In one group the concentration series used was matched across stimulation protocols (Exp3); thus, this group had matched total duration but more odor was delivered with the continuous stimuli. In the second group (Exp4), the concentration of the continuous stimuli was diluted to approximately 1/5^th^ of the pulsed stimuli. This lower concentration for the continuous stimuli should again approximate the amount of odor delivered in a 4 sec pulse train with a 10:40 ms duty cycle. In addition, to establish whether lowering the olfactometer outflow impacted relative detection threshold measures for either the pulsed or continuous protocols, the outflow velocity of the olfactometer was decreased from 80 cm/s (as used in experiments 1 and 2) to 30 cm/s in experiments 3 and 4. These four experimental groups could then be statistically compared.

One concern with interleaving clean and odor laden air, as was done in the above experiments, was that interleaving lacks the oscillation in air flow velocity, which occurs on a wing beat-to-beat cycle during odor guided flight [6]. Thus two experiments were designed to quantify the role that pulsing method has on measures of detection. In experiments 5 and 6 (Table 1) moths were tested with puffed stimuli (i.e. not interleaving clean air) relative to continuous stimuli. Here we used both the 30 cm/s and 80 cm/s olfactometer outflow velocities in separate groups. Both outflow velocities were used so that interactive effects of velocity with pulsing method (Exp. 1 and 3) could be statistically compared.

Finally, in all of the above experimental groups, odor was presented from low to high concentration in order to minimize non-associative effects associated with repeated exposures to the unrewarded CS. It is not known if these repeated stimuli have a substantial impact on the resulting concentration response function. Thus, a final experiment was designed to determine if repeated stimulations affected concentration response functions. In this case moths were conditioned and tested with 2-hexanone, but each sub group was tested with only a single concentration of the odor. Again on successive days, they were tested with either a 4000 ms pulse train (10:40 ms duty cycle) or an 800 ms continuous stimulation. This experimental group can be compared directly to the Exp1 subgroup where moths received the same odor but each moth was tested across the dilution series.

### Analysis

All analyses of behavioral data were performed in SAS using the general linear modeling (GLM) procedure. GLM has the advantage of hierarchically extracting variance components thereby allowing us to test effects of interest in a more parsimonious manner after removing variance associated with known effects. In all cases we analyzed behavioral data after subtractively accounting for responses to blank stimuli; this approach assumes only that false positive responses are an additive effect independent of the presence of odor. Each dataset was first individually analyzed to establish whether the concentration response function produced by the pulsed stimulus regime, resulted in enhanced detection relative to the continuous stimulus. Then, different experimental groups were compared to test for specific effects. Here variance attributable to individual differences, age and moth sex was extracted, all of which are well documented effects on CR probability [13,20-22]; hence, these effects are not shown but their significant interactions are, where relevant. We implemented a significance value of p<0.05 to identify significant effects. In all cases where post hoc analyses were performed, a Tukey’s Honestly Significant Difference (HSD) test was used with a standard test-wise error rate of p<0.05. Finally, MS Excel was used to plot all inset regression functions in figures. In all cases, we tested several functions for overall fit (as measured by R^2^) and parsimony. Thus, each displayed regression function represents the simplest model possible that best explains the increase in response across concentration.

## Results

### Brief 10 ms pulses optimize responsiveness of moths to odor (Exp 1 & 2)

Previous wind tunnel studies suggest that temporally structured odor plumes are not only necessary for odor-directed upwind flight [23], but that their details can be optimized to enhance plume tracking effectiveness [24,25]. Thus, our first objective was to determine whether the effect of duty cycle, the ratio of time a pulse is on vs. off, affects measures of detection. Data from Exp1 and 2 (Table 1) were used in this analysis, which collectively consisted of two duty cycles (10:40 ms and 40:10 ms on:off respectively) presented at 20 Hz, across three different odorants (HEX, HXL, and LOL). Three odors were used to test the generality of prior findings [8]. Results of the general linear model were based on an N of 360 moths (see Table 1). The overall model was significant (p<0.0001), and explained 32% of the variance in conditioned feeding response probability. 

**Figure 4:**
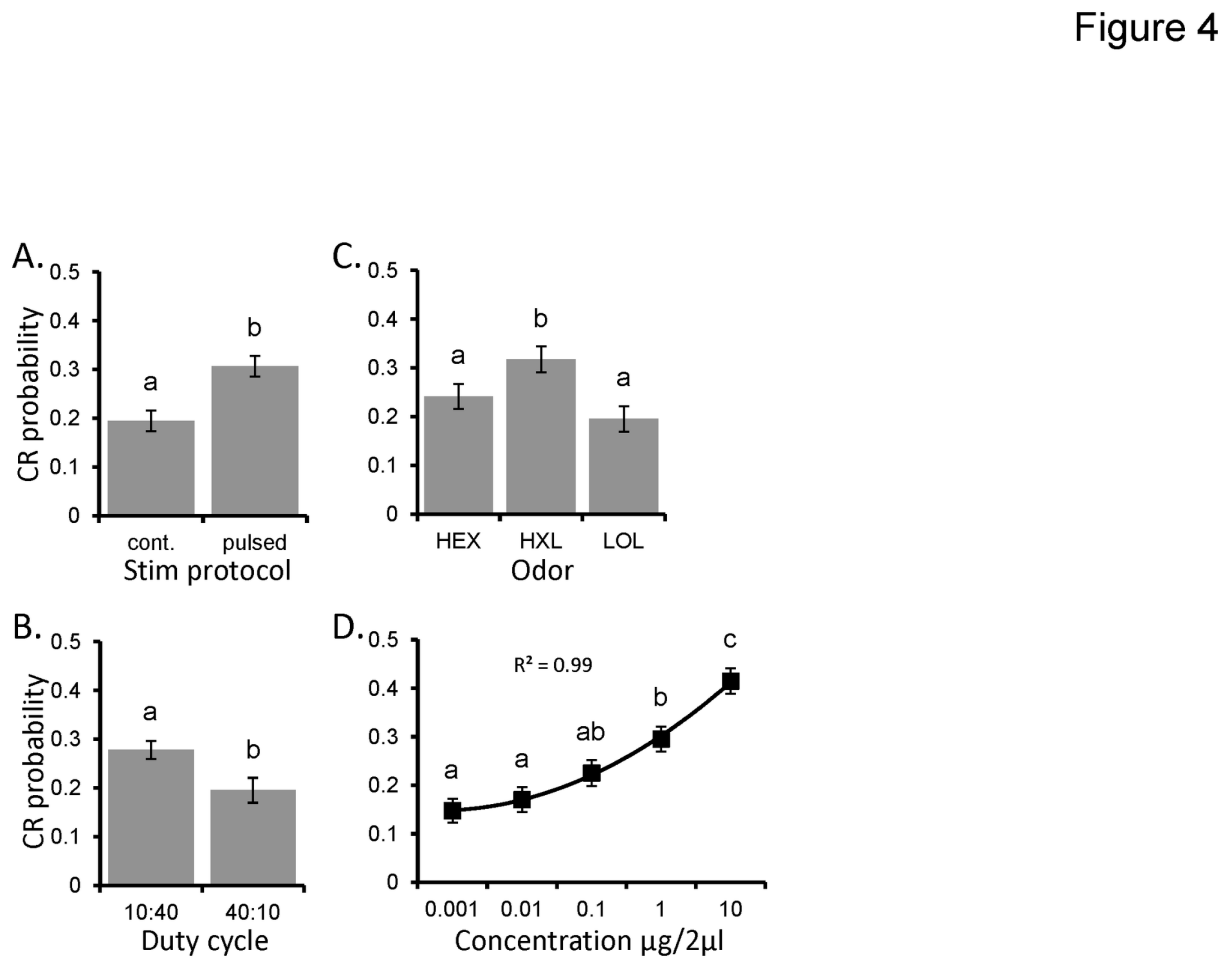
The features of olfactory stimuli and the context of delivery affect their detection. Main effects of: stimulus protocol (pulsed vs continuous; **A**); duty cycle (**B**); the odorant used (**C**); and stimulus concentration (**D**). In all panels the Y-axis represents conditioned response probability. Error bars represent +/-1 SE. Inset letters indicated significant differences between means based on a Tukeys HSD (p<0.05).

The main effects of stimulation protocol (pulsed vs. continuous), pulsing duty cycle, odorant, and stimulus concentration were all significant (p< 0.0001). Figure 4 displays the mean response probability as a function of these significant main effects. First, Figure 4A indicates moths were ~63% more likely to respond to odor when pulsed as opposed to presented as a continuous stimulus (CR probability = 0.19 and 0.31 for continuous and pulsed respectively). We also observed that moths exposed to the briefer 10:40 ms duty cycles were actually ~40% more likely to respond than those tested with 40:10 ms duty cycle (Figure 4B; CR probability = 0.28 and 0.20 for 10:40 ms and 40:10 ms respectively). Figure 4C displays the mean CR probability as a function of the main effect of the odorant used. Post hoc analysis (inset letters) indicates that moths responded to HXL significantly more than to HEX or LOL. Finally, as shown in Figure 4D, increasing stimulus concentration systematically increased the probability of eliciting a CR. Inset post hoc comparison of means indicates that the lowest three concentrations (0.001, 0.01 and 0.1) were not significantly different, though CR probability does trend upward with concentration in this range. Likewise the intermediate concentrations (0.1 and 1.0) were also not significantly different from each other. Nevertheless as absolute difference in comparisons between concentration are increased, differences become significant and always in a manner positively correlated with concentration. The inset second order polynomial (y = 0.0149x^2^ - 0.0234x + 0.1572) was the best fitting regression function, explaining 99.9% of the variance in mean CR probability as a function of concentration. Thus, with sufficient N, the concentration response function can be described as a curvilinear dose response curve.

**Figure 5:**
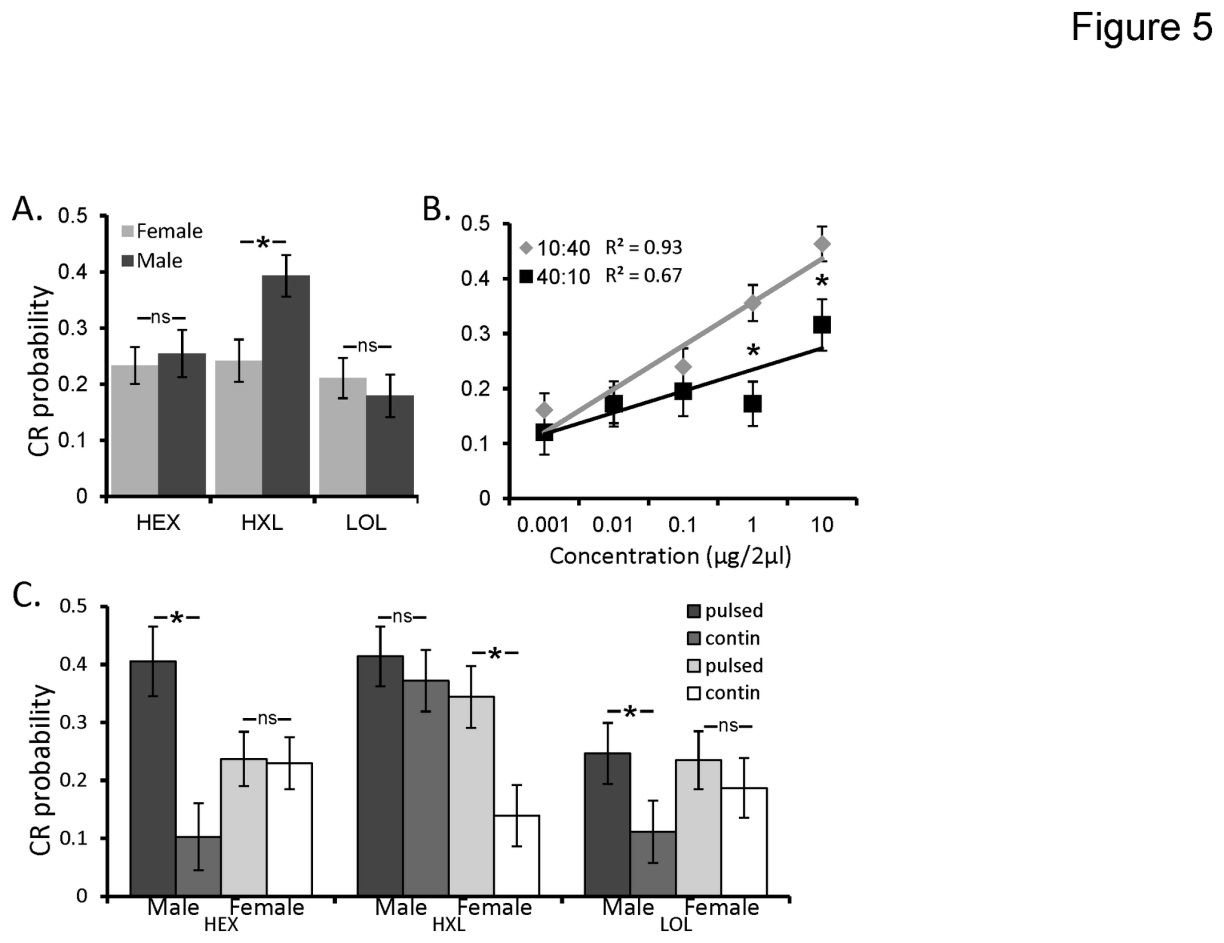
Stimulus parameters interact to affect CR probability. Post hoc analysis of the significant 2 and 3-way interactions of sex-by-odor (**A**); duty cycle-by-concentration (**B**); and sex-by-odor-by-stimulus protocol (**C**). In all panels the Y-axis represents conditioned response (CR) probability. Error bars represent +/-1 SE. Inset astrisks indicated significant differences between specific pairwise comparisons of means (Tukeys HSD; p<0.05).

Results of the GLM also indicate significant 2-way interactions between sex and odor (p=0.002), duty cycle and concentration (p=0.03), as well as a significant 3-way interaction between sex, odor and stimulation protocol (p<0.001). First, as shown in Figure 5A, the mean CR as a function of the interaction between sex and odor indicates that males and females produce idiosyncratic differences in CR probability to different odors. This is likely related to a combination of sex-dependent differences in sensitivity to some odors [20,26] as well as potential sex-dependent differences in responsiveness to a given odor. In this case, males were 63% more likely to respond to HXL than females (CR probability = 0.24 and 0.39 for females and males respectively). 

Figure 5B displays mean CR probability as a function of the significant interaction between duty cycle and concentration. Within concentration post hoc comparisons of differences in CR probability as a function of duty cycle indicates significant differences between the highest two concentrations (inset asterisks). Inset linear regression functions explain 93% of the variance in mean CR probability across concentration for the 10:40 ms duty cycle (y = 0.08x - 0.01) and 67% percent of the variance for 40:10 ms duty cycle (y = 0.03x + 0.03; note that polynomial regression functions did not increase R^2^ values). These regressions systematically diverge as concentration increases indicating different concentration response functions for the two duty cycles. This means that as concentration increases, brief 10 ms pulses are more likely to elicit a CR than the longer 40 ms pulses.

The significant 3-way interaction between sex, odor, and stimulation protocol (p<0.001) is shown in Figure 5C. Inset, are specific within-odor and sex post hoc comparisons to highlight that the pulsing effect appears to be both odor and sex dependent. For example while pulsing had a non significant effect on female CR probability for HEX and LOL, pulsing did result in a significant and substantial increase in CR probability for HXL. In males, however, pulsing resulted in significant differences in CR probability for both HEX and LOL but not HXL. Note that in all cases, there was a trend for pulsed stimuli to result in greater CR probability, highlighting the main effect of pulsing on olfactory sensitivity. 

Finally, we observed no significant interactions between odor and concentration (p>0.05) or stimulation protocol and concentration (p>0.05). Thus, while the intercepts were odor and stimulation protocol dependent (i.e. their main effects), the slopes of the respective concentration response functions were unaffected by either the odorant used or the stimulation protocol used. This means that pulsing odor shifts concentration response functions. Overall, these results imply that pulsing enhances sensitivity and brief 10 ms pulses are better than longer 40 ms pulses, particularly as concentration increases.

### The velocity of the stimulus impacts measures of detection; pulsing method does not (Exp 3-6)

Although we have matched the pulse frequency and stimulus flow velocity to the approximate the upwind speed of an actively tracking moth (Daly, unpublished observations), by interleaving clean air with odor laden air we impose a period during the pulse cycle where the olfactometer is facilitating active clearing of odor from the antennae. This approach reflects continuous movement in flight but may affect the time odor is retained by the antennae and hence affect the olfactory sensory interaction [27]. Therefore, keeping in mind that the moths are positioned in a constant exhaust flow, we tested whether changing the method of pulse delivery, by removing the interleaving of clean air between odor pulses, impacts detection measures. In addition, the output velocity from the olfactometer in the above behavioral experiment (80 cm/s) is within the approximate range of odor-guided flight speeds. However, modeling studies predict that if air flow drops below a critical level, the odor laden air will cease to penetrate the antennal sensillar array [2,28].

Therefore, to assess the effects of both interleaving clean air between individual pulses, and the olfactometer outflow velocity, we compared four different experimental groups of moths (Table 1, Exp 3-6; N=60 moth/group) in a 2-way factorial design. In this case, after confirming that differences between pulsed and continuous stimuli were the same in these groups, we then dropped responses to continuous stimuli and reanalyzed using only responses to pulsed stimuli. All groups were conditioned to respond to HEX and then tested with either an 80 cm/s or 30 cm/s stimulus that was either interleaved with clean air or puffed without interleaving. 

**Figure 6:**
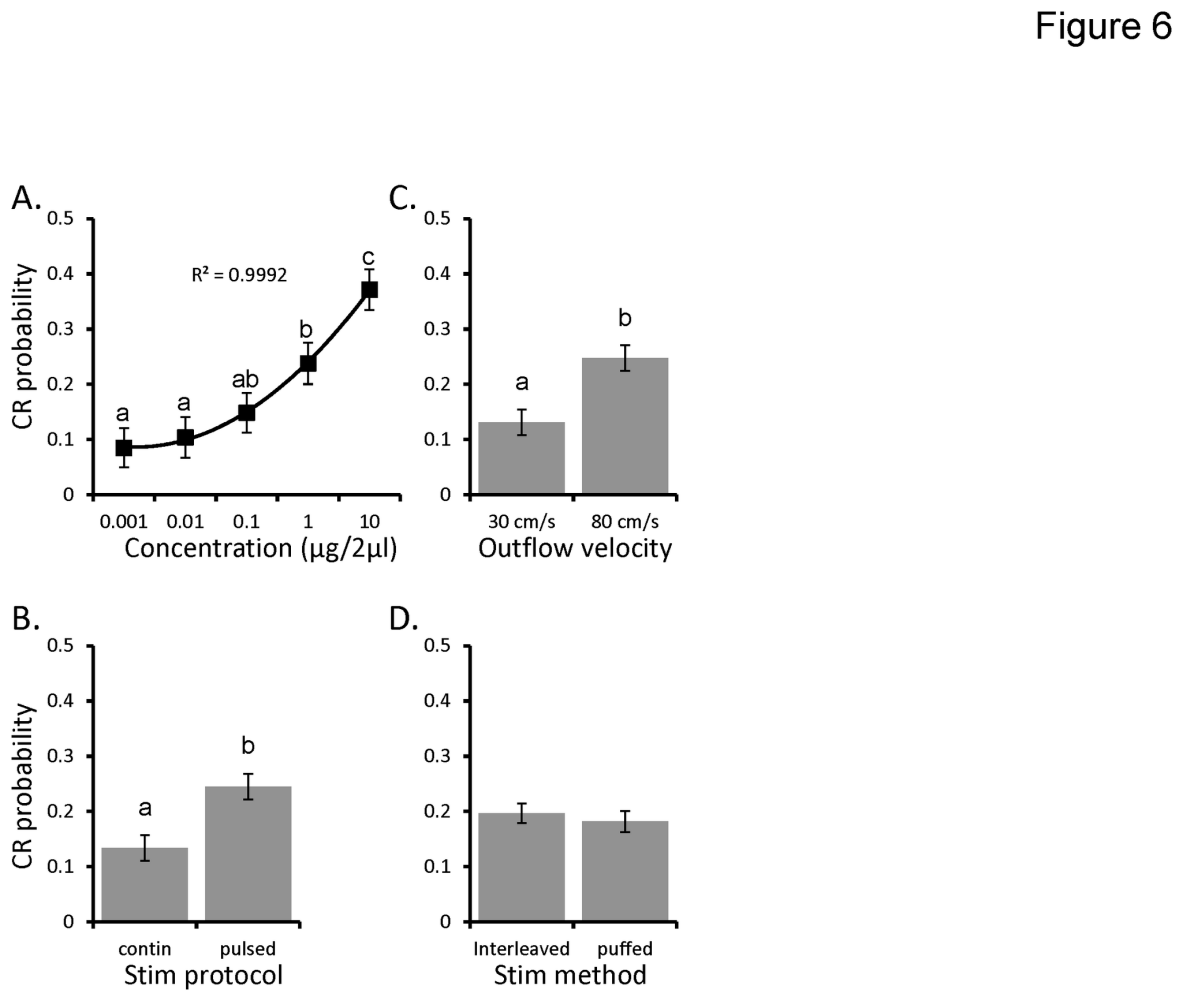
Pulsing method does not affect CR probability, olfactometer flow velocity does. Mean CR probability as a function of: concentration (**A**); stimulation protocol (**B**); olfactometer outflow velocity (**C**); and pulsing method (interleaved vs puffed; **D**). In all panels the Y-axis represents conditioned response (CR) probability. Error bars represent +/-1 SE. Inset letters indicated significant differences between means (Tukeys HSD; p<0.05).

The overall GLM was significant (p<0.001), explaining 38% of the variance in CR probability. We again observed significant main effects of concentration and stimulus protocol (p<0.001; see Figures 6A and B respectively). The main effect of the velocity of the odor was also significant (p<0.001). Figure 6C indicates that with the higher velocity, moths were 89% more likely to elicit a conditioned feeding response (CR probability = 0.13 and 0.25 for 30 cm/s and 80 cm/s respectively). However, the main effect of pulsing method (whether puffed or interleaved) was not significant (p>0.05; Figure 6D). It is worth noting that there were no significant higher order interactions. Thus, increased velocity results in increased responsiveness to the conditioning odor across the concentration series; this is possibly due to flow velocity-dependent boundary layer effects around the sensillum [2,28]. However, within the context of the ambient exhaust flow of 30 cm/s, oscillating the olfactometer outflow by puffing did not impact measures of odor detection relative to interleaving.

### Non-associative effects do not impact concentration response functions (Exp 7)

The details of the concentration response functions could be affected by repeated exposure to test stimuli; these effects could furthermore be unique to the specific stimulus protocol. For example, in addition to sensory adaptation, extinction of the conditioned feeding response or sensitization to either olfactory or non-olfactory components of stimuli could also occur. If animals perceive the two stimulation protocols as different in intensity, this could lead to differences in rate of extinction across successive test trials. Of particular concern in the present study is that pulse trains may represent a more intense stimulus, specifically in terms of non-olfactory, possibly mechanosensory effects (see Figures 2 and 3); this potential sensitizing effect could differentially impact measures of detection. Therefore, in this experiment we controlled for the effects of repeated stimulation by testing animals with a single stimulus (either a pulsed or continuous stimulus) at a single concentration (Table 1, Exp7). Thus, each moth received a blank test followed by a single odor stimulus at one concentration per day, thereby eliminating any within-day non-associative effects. Each concentration is therefore represented by different subgroups of animals but we maintained a within animal design for each concentration. We compared this experimental group with an experimentally matched group where moths were tested across concentrations (Exp1, HEX subgroup only). 

**Figure 7:**
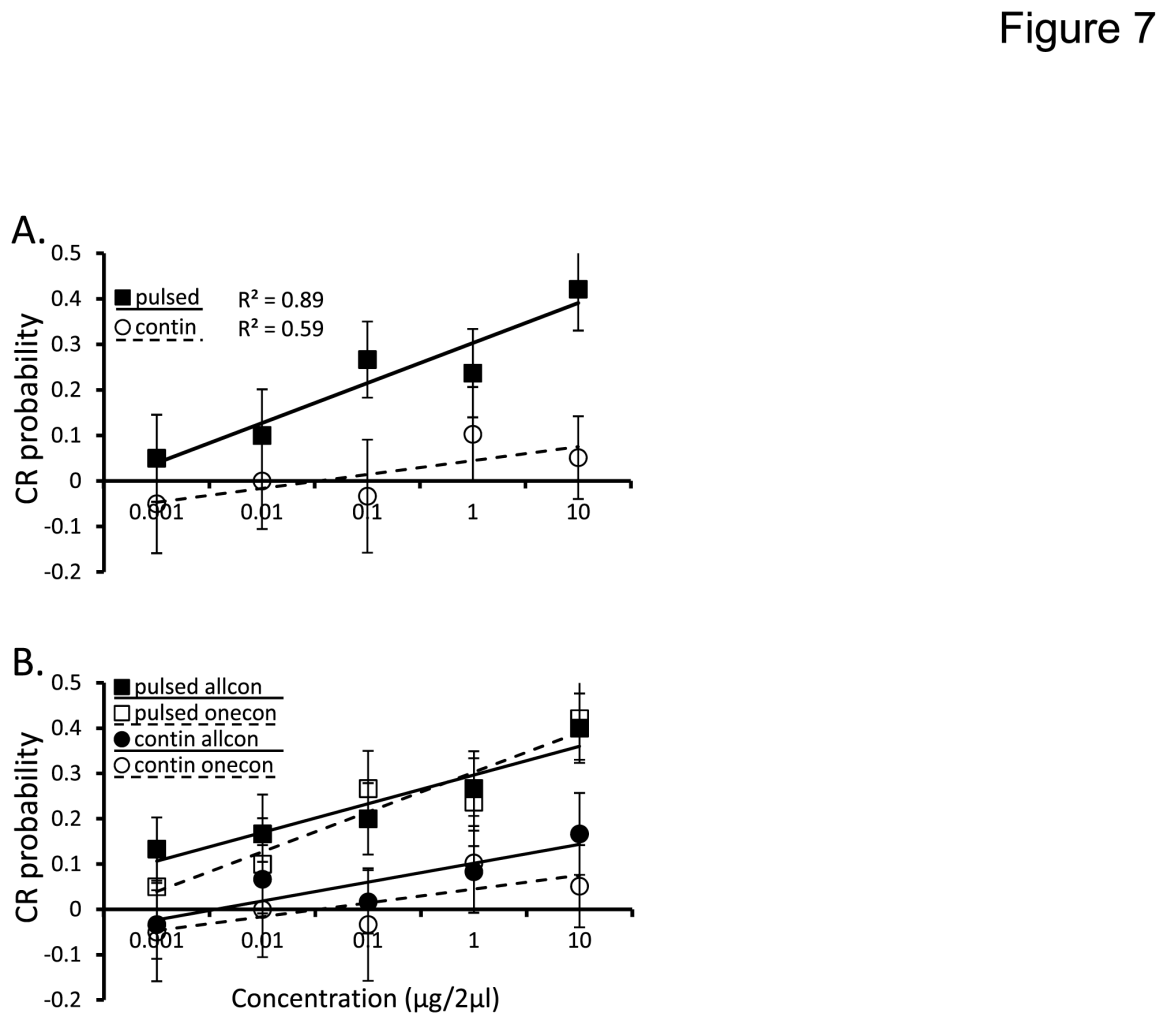
Non-associative effects do not affect concentration response functions. Mean CR probability as a function of the non-significant interactions of stimulation protocol-by-concentration (**A**); test method by stimulation protocol by concentration (**B**). Inset are linear regression lines for each condition. Here again, the only the significant main effects of concentration and stimulation protocol are evident.

The overall results of the GLM were significant (p=0.004) explaining 57% of the variance in conditioned feeding responses. We again found the same pattern of significant main effects of concentration (not shown) and stimulation protocol (p<0.0001 respectively). Figure 7A shows the significant main effect of the stimulation protocol. We present these results as a function of concentration, to highlight the non-significant interaction between stimulation protocol and concentration (p=0.589). This non-significant interaction indicates that CR probabilities for pulsed and continuous stimuli increased at approximately the same rate across concentration and only significantly differ in their Y-axis intercepts (i.e. the main effect of stimulation protocol). However, in this case our primary interest was the effect of test method (i.e. moths that received a single test concentration versus those tested across all concentrations); this effect was not significant (p=0.604), nor was its interaction with concentration (p=0.826). These results imply that there was no net impact of non-associative effects such as sensory adaptation, sensitization or extinction. Furthermore, the test method-by-stimulation protocol interaction was also not significant (p=0.755) indicating that CR probability to pulsed and continuous stimuli was the same independent of test method. In fact, as Figure 7B shows, when broken down by whether moths were tested with all concentrations or just one, the response to each concentration was approximately the same as well as the slope of the regression functions. Thus, the effect of stimulation protocol (pulsed versus continuous) cannot be explained as the result of differences in non-associative effects. 

### Matched stimulus duration does not enhance responsiveness to continuous stimuli (Exp 3 & 4)

In the above experiments, the total integrated stimulus-on-time was matched, which meant that the duration of the pulse trains (4000 ms) were always longer than the continuous stimuli (800 ms and 3200 ms for the 10:40 ms and 40:10 ms duty cycles respectively). While matching total stimulus-on-time corrects for the approximate amount of odor delivered between the two stimulus protocols, it does so at the cost of having different stimulus durations. To assess stimulation time as a potential confound, we conducted two experiments. In both, we used 4000 ms stimuli for both the pulse train and continuous stimuli. In the first case, the concentrations of the continuous stimuli were the same as for the pulsed (Table 1, Exp3); in the second, the concentration of the continuous stimuli was reduced to 1/5 of the pulsed concentration, thereby resulting in approximately the same time-integrated amount of odor delivered as in the 10:40 ms duty cycle pulse trains (Table 1, Exp4). 

**Figure 8:**
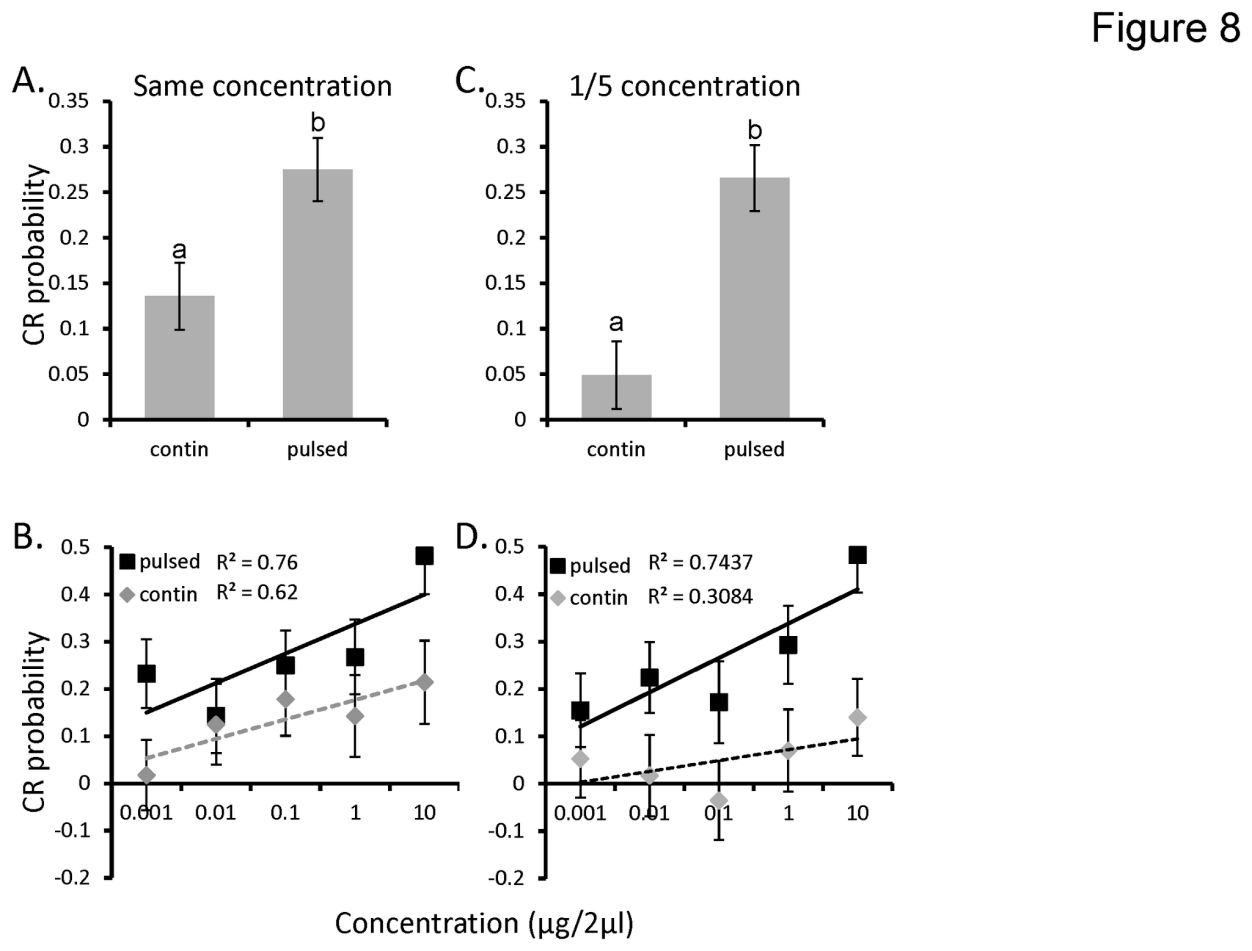
Differences in stimulus duration do not account for differences in pulsed versus continuous stimuli. Main effect of stimulus protocol (pulsed vs continuous) for odor stimuli that were presented for the same duration either and the same concentration (**A**, **B**); or concentration corrected to 1/5^th^ to match the total odor delivered (**C**, **D**). In panels **B** and **D**, the main effect of stimulation protocol for each approach, is shown as a function of concentration to highlight that rates of increase in CR probability as a function of concentration are not significantly different. The Y-axis represents conditioned response (CR) probability. Error bars represent +/-1 SE. Inset letters indicate significant differences between means (Tukeys HSD; p<0.05).

Results of the GLM analysis where moths were tested with the same duration and concentration was significant (p<0.001) accounting for 44% of the variance in CR probability. As above, odor concentration was significant (p=0.004). After accounting for concentration we again found the main effect of stimulation protocol was significant (p=0.001) and its interaction with concentration was not (p=0.374). Figure 8A displays the mean CR probability as a function of stimulation protocol and again shows that pulse stimuli produced twice the CR probability as continuous (0.14 and 0.28 respectively for continuous and pulsed). Figure 8B breaks this effect down by concentration and the near parallel inset linear regressions highlight the non-significant concentration by stimulation protocol interaction.

By comparison, we observed the same pattern when we tested moths with the same duration and reduced odor concentration to 1/5 (Figures 8C and 8D). The GLM model for this experimental group was significant (p<0.0001) accounting for 32% of the variance in CR probability. We again found that CR probability was significantly lower for continuous stimuli (p<0.0001). As shown in Figure 8C, this represents only ~18% of the CR probability driven by the pulsed odor. Finally, the interaction between stimulation protocol and concentration was again not significant (p=0.5343) indicating that the difference between stimulation methods had little effect on the concentration response function. 

### Differences in false positive responses accounts for the pulsing-dependent enhanced sensitivity (Exp 1-7)

**Figure 9:**
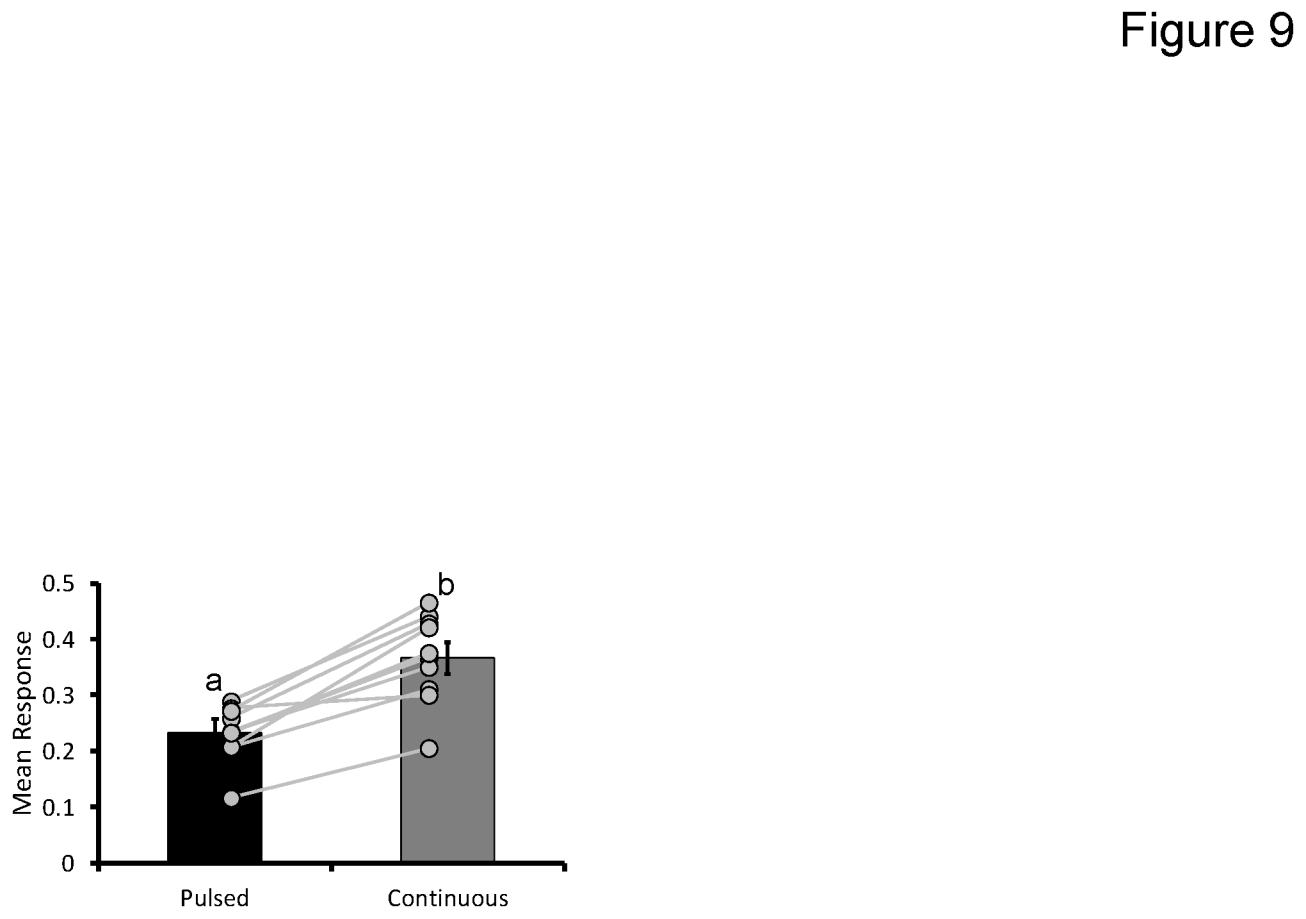
Enhanced sensitivity to pulsed stimuli is the result of lower false positive rates. t-test comparison of blank responses elicited by the pulsed vs continuous stimuli. The Y-axis represents response probability and inset letters indicate significant differences between means based on a Tukeys HSD (p<0.05). Inset lines show mean differences between pulsed and continuous blanks by experimental subgroup (N = 10 sub-groupings).

As mentioned above, the two stimulus protocols differ, not only in the temporal structure of the odor delivered, but they also contain different amounts of non-olfactory artifacts (see Figure 2). These artifacts are manifest as oscillating spikes and dips in flow rate, which are caused by the valve actuation; this was particularly notable with higher olfactometer flow rates. As Figure 2 demonstrated, while the continuous stimulus contains this artifact at the beginning and end of the stimulus, the pulse trains have one at the beginning and end of every pulse in the 4 s train. Therefore, to correct for responses that might be driven by these artifacts we collected blanks for each corresponding stimulus as a correction method. To assess the differences in blank responses we ran a final analysis using all of the blank responses from all of the experimental data available (N = 750 blank tests for pulsed and continuous stimuli respectively). Contrary to our expectations, the results of an omnibus paired t-test between pulsed blanks and continuous blanks across all experimental groups indicated that pulsed blanks produced significantly lower response rate than did continuous blanks (p<0.0001). As shown in Figure 9, even when broken down into the 10 individual sub groups of data for the different experiments, all groups showed this same general trend; continuous blank stimuli are on average 1.5 times more likely to elicit a response than pulsed blank stimuli (CR probability = 0.24 and 0.38 for pulsed and continuous blanks respectively).

## Discussion

The goal of this study was to determine how the parameters of odor pulse trains affect measures of odor detection in moths that were previously conditioned to respond to a target odor. In addition to confirming that pulsed odor is generally more easily detected, we found that briefer and higher velocity stimuli enhanced measures of detection. The sensitivity to pulsed stimuli was dependent on the interaction of the odor used to condition and test the moths as well as the moth’s sex; this is entirely consistent with prior findings [29-31]. Finally, we demonstrate that the pulsing-dependent enhanced sensitivity is largely attributable to lowered false positive rates elicited by pulsed stimuli; that is, moths are less likely to respond to a blank pulse train than a continuous blank. This latter finding is completely novel to our knowledge and surprising given the anemometry results demonstrating that the non-olfactory components of the pulsed stimulus were considerably greater. Overall, this pattern of results suggests that the olfactory system of the moth has adapted to detect odor specifically under conditions of rapid and transient exposures to odor and this may be related to the impact that wing beating naturally has on the olfactory system [6,7,28,32].

Clean air is an important component of the odor experience and necessary for normal odor-guided flight to occur [23-25]. Periods of clean air reduce sensory adaptation effects and provide information about the boundary of odor plumes; without periods of clean air moths are simply unable to track plumes [23]. Along with sensory adaptation [24], prolonged stimulation can result in tonic activation of receptor cells, thereby reducing the ability of ORNs to follow fluctuations in odor concentration. We note that sensory adaptation was not an issue in our study; moths were always presented with no more than 6 trials of odor within a given day, odor was presented from lowest to highest concentration, and we used 6 min inter-trial intervals. Furthermore, our control tests, which specifically tested for non-associative effects, established that non-associative effects were not present at a detectable level. 

Large scale details plume structure such as plume width are an important components that shape odor guided flight [33]. Nevertheless fine scale structure, such brief rapid odor pulses, reduce the time required by moths to find an odor source in wind tunnel studies [24]. Thus it appears that the olfactory system of these plume tracking moths are optimized to respond to extremely brief stimuli with interstitials of clean air. Our findings support these claims and suggest that the temporal resolution is finer than previously thought. It is worth noting that several studies characterizing the frequency response from several moth species did not observe tracking at these higher frequencies in either the antennae [10,11] or antennal lobe [34]. These latter studies all used relatively long pulse durations (i.e. 50 ms), increasing pulse train frequency by decreasing the off time. Indeed by comparison, the 40:10 ms duty cycle, which most closely matches the above mentioned studies, also produced weaker EAG oscillations and higher detection thresholds. Thus, when considered within the context of prior results, the current study suggests that relatively longer stimulations with brief inter pulse intervals negatively impacts the sensitivity of the antennae, possibly due to sensory adaptation. 

Detection thresholds were also enhanced when odor was presented at a relatively higher velocity. The two velocities we used were selected to fall in the range of typical plume tracking behavior [35]. Baker et al. [35], showed that as wind speeds are lowered, moths exhibit greater difficulty tracking plumes to the source; in zero wind conditions the ability of male moths to find pheromone sources dropped to 20%. These researchers suggest that the disruption of plume structure was the main factor in reducing plume tracking effectiveness. In addition, we again highlight that airflow velocity is also critical for air and odor to effectively penetrate the sensillar array [28]. It is also worth noting that the effect of increased velocity lowering detection thresholds is consistent with the hypothesis that olfactory receptor cells (and the sensilla in which they are housed), collectively act as flux detectors [12,36]. However, unlike studies which directly test the flux hypothesis through measurement of receptor neuron responses at different flow velocities [37], we measure behavior. Behavior is the culmination of both sensory transduction events as well as several layers of post transduction processing. Thus, it is difficult to directly relate our findings to this hypothesis.

Perhaps the most striking finding of the current study is that moths were significantly less likely to respond to blank pulse trains as opposed to a continuous blank. Given that pulse trains produce a considerably greater non-olfactory cue as measured by anemometry, this result seems both surprising and counter intuitive. However, we note that this effect was observed in every experimental group and independently by all three observers involved in collecting behavioral data. The fact that this moth species is adept at apparently ignoring periodically structured airflow artifacts provides a tantalizing hint at the possibility of an evolutionary adaptation, at the level of sensory processing. We speculate that the AL, at some level, may be “expecting” (or is otherwise tolerant of) such artifacts under the real world circumstances of flight. This might be coordinated within the antennal lobe via direct input from ascending centrifugal cells emanating from the mesothoracic ganglia where motor programs for flight are generated [38]. Analogous findings have been described in several other species and sensory domains [39,40]. For example, it has been shown that a corollary discharge from the mesothoracic ganglion of cricket CPG involved with song production, projects to the primary auditory center in the prothoracic ganglion and prevents self-generated sound from saturating the auditory system [39]. While it remains to be determined how this anatomical connectivity between flight sensory-motor centers and the primary olfactory center functions, the fact that the olfactory system seems adept at accurately responding to pulsed stimuli in the wing beat frequency range, suggests that a similar type of system has evolved here.

In conclusion, it has long been known that insects actively track odor plumes from a point of initial detection to its source using optomotor anemotactic flight behaviors [35,41-43], as well as brief surges of forward flight [44]. As we have pointed out, odor-guided flight requires intermittency of odor to successfully zigzag back and forth through the plume as the insect progresses to the source [25]. Furthermore, any wing beating related effects on airflow are superimposed on this zigzagging behavior. By analogy, ground-scent tracking animals, including humans, also take an indirect, essentially zigzagging track, as they follow a scent trail [45,46]. Sniffing, like wing beating, is superimposed on this zigzagging locomotory pattern, although there are differences in the details. Each respiration cycle in mammals drives a response in the olfactory epithelium and hence the OB and olfactory cortex [3,47]; in both taxa this occurs even in the absence of an odorant [8,48]. In insects, it has only recently been shown using EAG that the antennae of different species track odors pulsed at such high frequencies [8,49-51]. Nevertheless, periodically structured plumes at or near typical wing beat frequencies have been shown to be optimal for rapid in-flight source location [24] further hinting at the importance of temporally structured stimuli and the proposition that the time domain is reserved for encoding time varying concentration [9,52]. At the level of the first synaptic neuropil of the olfactory system, it has been demonstrated that neurons intrinsic to primary olfactory neuropil can be entrained to odors delivered at periodic frequencies consistent with sniffing [47] and wing beating [8]. Finally, as with the moth [38], there are centrifugal projections from brain stem regions involved with respiratory pattern generation to the rat olfactory bulb [53,54]. This overall pattern of continuity, which spans from neural architecture, to functional response, to behavior, suggests an evolutionarily convergent solution for animals to experience the fine spatiotemporal structure of the chemical environment. 
